# Point Mutations in the Glycoprotein Ectodomain of Field Rabies Viruses Mediate Cell Culture Adaptation through Improved Virus Release in a Host Cell Dependent and Independent Manner

**DOI:** 10.3390/v13101989

**Published:** 2021-10-03

**Authors:** Sabine Nitschel, Luca M. Zaeck, Madlin Potratz, Tobias Nolden, Verena te Kamp, Kati Franzke, Dirk Höper, Florian Pfaff, Stefan Finke

**Affiliations:** 1 Friedrich-Loeffler-Institut (FLI), Federal Research Institute for Animal Health, Institute of Molecular Virology and Cell Biology (IMVZ), 17493 Greifswald-Insel Riems, Germany; bine.nemitz@freenet.de (S.N.); luca.zaeck@fli.de (L.M.Z.); madlinpotratz92@gmail.com (M.P.); tobias.nolden@boehringer-ingelheim.com (T.N.); verena.tekamp@gmail.com (V.t.K.); 2 Friedrich-Loeffler-Institut (FLI), Federal Research Institute for Animal Health, Institute of Infectiology (IMED), 17493 Greifswald-Insel Riems, Germany; kati.franzke@fli.de; 3 Friedrich-Loeffler-Institut (FLI), Federal Research Institute for Animal Health, Institute of Diagnostic Virology (IVD), 17493 Greifswald-Insel Riems, Germany; dirk.hoeper@fli.de (D.H.); florian.pfaff@fli.de (F.P.)

**Keywords:** rabies virus, cell culture adaptation, virus release, field virus, glycoprotein

## Abstract

Molecular details of field rabies virus (RABV) adaptation to cell culture replication are insufficiently understood. A better understanding of adaptation may not only reveal requirements for efficient RABV replication in cell lines, but may also provide novel insights into RABV biology and adaptation-related loss of virulence and pathogenicity. Using two recombinant field rabies virus clones (rRABV Dog and rRABV Fox), we performed virus passages in three different cell lines to identify cell culture adaptive mutations. Ten passages were sufficient for the acquisition of adaptive mutations in the glycoprotein G and in the C-terminus of phosphoprotein P. Apart from the insertion of a glycosylation sequon via the mutation D247N in either virus, both acquired additional and cell line-specific mutations after passages on BHK (K425N) and MDCK-II (R346S or R350G) cells. As determined by virus replication kinetics, complementation, and immunofluorescence analysis, the major bottleneck in cell culture replication was the intracellular accumulation of field virus G protein, which was overcome after the acquisition of the adaptive mutations. Our data indicate that limited release of extracellular infectious virus at the plasma membrane is a defined characteristic of highly virulent field rabies viruses and we hypothesize that the observed suboptimal release of infectious virions is due to the inverse correlation of virus release and virulence in vivo.

## 1. Introduction

Rabies virus (RABV) and related lyssaviruses are highly neurotropic rhabdoviruses, which cause an almost invariably fatal disease once neurological symptoms appear [1]. The negative sense ~12 kb RNA genome of RABV encodes for the five virus proteins: N (nucleoprotein), P (phosphoprotein), M (matrix protein), G (glycoprotein), and L (large polymerase). N, P, and L are essential for RABV replication and transcription. M and G are essentially involved in infectious virus particle release through budding at the plasma membrane [2]. 

The RABV glycoprotein G is located at the surface of RABV particles and is responsible for receptor binding and ribonucleoprotein (RNP) release in the cytoplasm by pH-dependent membrane fusion. Although essential for cell entry and in vivo spread [3], G is dispensable for virus particle release, resulting in the production of non-infectious particles in absence of G. However, for the production of infectious virus particles, G is an essential component and is simultaneously increasing the M-dependent budding efficiency [4,5]. The ectodomain of the RABV G protein is comprised of the fusion domain (FD), the pleckstrin homology domain (PHD), and the central domains (CD), which switch from a bent hairpin to an extended conformation by basic-to-acidic pH change [6].

Neuronal cell adhesion molecule (NCAM1), p75 neurotrophin receptor (p75NTR), nicotinic acetylcholine receptor (nAChR), and metabotropic glutamate receptor subtype 2 (mGluR2) have been described as RABV entry receptors [7,8,9,10]. In primary neurons, entry of co-internalized p75NTR and RABV particles into the retrograde axonal transport machinery has been directly demonstrated [11], suggesting a role of p75NTR as a receptor in neuroinvasion. While additional receptors may exist [12], it is still not known whether and to what extent the individual receptor molecules contribute to in vivo infection, spread in the nervous system, and limited virus replication of non-cell line-adapted field virus isolates. In cell culture, the broad panel of non-neuronal cell lines susceptible to laboratory RABV strains indicates the existence of more ubiquitous receptors, which support virus infection. Since field RABV isolates usually replicate poorly in cell culture [13,14], many cell culture-based approaches in RABV research include fixed viruses, which are adapted for improved replication by serial passages either in cell culture or non-natural host animals. 

Adaptation of field RABV isolates to more ubiquitous non-neuronal receptors may represent one key bottleneck to successful RABV cell culture replication. However, there is no experimental proof available that receptor usage differs between field and laboratory RABVs. By demonstrating infection of CNS-resident astrocytes by different field RABVs, a recent comparison of various field and lab-adapted RABV strains revealed an even broader host cell tropism of field RABVs in vivo [15]. 

Beyond receptor usage, infection of glial cells by highly virulent field RABVs may also be determined by the replication potential and the capacity of the virus to antagonize the innate immune response in that specific cell type [15,16]. The abortive infection of astrocytes in the CNS of mice infected with lab-adapted RABV as the result of a strong astrocyte-mediated type I interferon expression [17] support the hypothesis that cell type-specific antiviral responses can be decisive for host cell tropism of RABV in vivo.

Another receptor-independent factor is the glycosylation status of the surface glycoprotein G. Typically, in the course of rabies virus cell culture adaptation, the acquisition of additional N-glycosylation sites in the surface glycoprotein G leads to a more efficient G expression [18,19,20,21], which led to the hypothesis that virus production by an increased G gene expression might play a major role in cell culture adaptation. Accordingly, Yamada et al. [20] showed that additional glycosylation increased the release of infectious virus via complementation of G-deficient RABV, indicating that indeed limitations in infectious virus release can be an important factor in RABV cell culture replication and adaptation of field viruses. 

The availability of recombinant field RABV clones [13] allowed us to perform in vitro cell culture adaptation experiments with field viruses derived from fox and dog isolates. Starting with genetically defined field virus clones, serial passaging on three different cell lines was performed to identify cell culture adaptive mutations, to assess virus- and cell line-specific constraints during adaptation, and to gain an insight into the mechanisms of limited field virus cell culture replication. The identification of involved mutations and their impact on infectious virus release, together with the discussion of possible effects on field RABV virulence and pathogenicity, lead to a more detailed understanding of field RABV biology and provide new insights into RABV pathology mechanisms and virus attenuation. 

## 2. Materials and Methods 

Cells and viruses. BSR T7/5 baby hamster kidney (BHK) cells [22], murine neuroblastoma cells (Na 42/13), and canine epithelial cells (Madin-Darby Canine Kidney; MDCK-II) were provided by the Collection of Cell Lines in Veterinary Medicine (CCLV; reference numbers 0583, 0228, and 1061, respectively), Friedrich–Loeffler-Institut Riems, Germany. Recombinant rRABV-DogA14 and rRABV-Fox9 [13], referred to as rRABV Dog and rRABV Fox here, are recombinant clones of street RABVs isolated from a dog (Azerbaijan 2002; rabies virus strain 5989/lib205/12) and a fox (Germany 1998; rabies virus strain 148/lib01041), respectively [23]. The virus cDNA sequence in plasmid prRABV-DogA14 was 100% identical to the progenitor virus consensus sequence (accession number LN879480) and the cDNA sequence in rRABV-Fox9 exhibited one amino acid substitution in the N at position 23 when compared to the progenitor fox virus isolate (accession number LN879481). SAD ΔG GFP is a derivative of the attenuated vaccine virus clone SAD L16 [24], in which the G-coding sequence was replaced by a sequence coding for the green fluorescence protein (GFP) [25].

Virus stock production and passages. After rescue of the recombinant RABVs (rRABVs) on the BHK cell clone BSR T7/5, rRABV Dog and rRABV Fox [13] stocks were produced by five passages on Na 42/13 cells. For serial passages on different cell lines, BHK (BSR T7/5), Na 42/13 and MDCK-II cells were initially infected at a multiplicity of infection (MOI) of 0.01 (infectious virus titers were determined on Na 42/13 cells). Every third day, 200 to 500 µL of the cell culture supernatant were transferred to freshly seeded cell cultures. Infection and spread of the cells were monitored by indirect immunofluorescence against RABV nucleoprotein N in separate dishes infected in parallel. SAD ΔG GFP virus stocks were generated on the MGon packaging cell line, which is a BSR cell clone that expresses the RABV M and G proteins after induction with doxycycline [26]. 

Generation of rRABV Dog-NTN and BSR T7/5-free rescue of rRABV Dog*. The full-length plasmid of rRABV Dog mutant rRABV Dog-NTN (D247N, A400T, and K425N amino acid exchanges in G) was generated by sequential site-directed mutagenesis of the full length cDNA plasmid prRABV-DogA14 [13]. After insertion of the D247N mutation with the primer pair 5′-GAT GGA ACA TGG GTC GCC ATG CAA ACA TCA AAT GAG AC CA AAT-3′/5′-GAC CCA TGT TCC ATC CAT AAG TCT A-3′, the resultant prRABV Dog-D247N clone was used for the insertion of the K425N mutation with the primers 5′-TGT TGA GGT TCA CCT TCC GGA TGT GCA CAA TCA GGT CTC AG-3′ and 5′-AGG TGA ACC TCA ACA AAA TCC TCA G-3′ to yield prRABV Dog-D247N/K425N. The final cDNA clone prRABV Dog-NTN was generated by insertion of the A400T mutation in prRABV Dog-D247N/K425N with the primers 5′-CTG ATG CAC CCC CTG ACG GAT CCG TCC ACA GTT-3′ and 5′-CAG GGG GTG CAT CAG GGG AAT AAC T-3′. Recombinant virus rRABV Dog-NTN was rescued from the cDNA full length clone plasmid by transfection of BSR T7/5 cells as described previously [13], and the sequence of the resultant stock virus was confirmed by Sanger sequencing of RT-PCR products.

In order to avoid any BHK-specific adaptation, rRABV Dog* was directly rescued in Na 42/13 cells by co-transfection of prRABV-DogA14 with plasmids coding for RABV N, P, L [27], and codon-optimized bacteriophage T7 RNA polymerase [15]. Five days post-infection, Na 42/13 cells were split at a ratio of 1:3 to two 3.5 cm^2^ wells and presence of the virus was monitored by indirect immunofluorescence with RABV N- and G-specific antibodies. Cell culture supernatants of positive samples were transferred to freshly seeded Na 42/13 cells for stock virus production. The absence of adaptive mutations in G was verified by Sanger sequencing of RT-PCR products.

DNA transfection. Plasmid DNA transfections into BSR T7/5 cells were performed with polyethyleneimine (PEI; Sigma–Aldrich, Burlington, MA, USA). Briefly, 2.5 to 5.0 × 10^5^ cells were seeded in 3.5 cm dishes. After overnight incubation, the cell culture medium was replaced by 1 mL serum-free DMEM. To prepare the transfection mix, plasmid DNA and PEI were separately diluted in 400 µL DMEM each and then mixed after a 5 min incubation at room temperature. After an additional incubation of 20 min and 1 h after exchanging the cell culture medium for serum-free DMEM, the mix was added to the cell culture. The cell culture medium including the transfection mix was exchanged for fresh culture medium after 3.5 h incubation at 37 °C.

In trans-complementation of G-deficient RABV. G gene-deleted SAD ΔG GFP was complemented by plasmid-expressed G protein as described previously [28]. Briefly, 3 × 10^6^ BSR T7/5 in a T25 cell culture flask were infected at an MOI of 1. After 2 h incubation at 37 °C, the cells were trypsinized and transferred to 6-well cell culture plates. After an over-night incubation, the infection was monitored by GFP autofluorescence and the plasmid DNA was transfected. Two days later, the cell culture supernatants were collected, centrifuged (5 min, 1200× *g*), and the infectious virus titers in the supernatants were determined by titration on BSR T7/5 cells. 

RNA extraction, RT-PCR, and cloning of G gene cDNAs. RNA was prepared from infected cell monolayers with TRIzol^TM^ Reagent (Invitrogen) according to the supplier’s instructions. Four hundred nanograms of RNA were used in a reverse transcription (RT) with 100 U RevertAid Premium Reverse Transcriptase (Thermo Scientific) according to manufacturer’s instructions. The 1.6 kb G-coding cDNAs of rRABV Dog variants passaged on BSR T7/5 cells were cloned in a plasmid vector by PCR amplification of the respective G-coding sequence with the primers 5‘-TAA TCA GAA TTC GGA AAG ATG GTT CCT CAG GTT CTT TTG-3‘ and 5‘-ATC TAT GCT AGC GAA GCG TCG AGA GGA TGA CC-3‘, and insertion of the EcoRI/NheI-digested PCR DNA fragment in an EcoRI/NheI-digested pCAGGS vector [29].

Full length RABV sequencing. Total RNA was extracted from cell culture supernatant using peqGOLD TriFast (Peqlab, VWR International GmbH). Full-length virus genome sequencing was done as described before [30]. Briefly, double-stranded cDNA was generated using a cDNA synthesis system kit (Roche) and random hexamer primers. For library preparation of full-length genome cDNA clones and parental isolates, 1 μg double-stranded cDNA were fragmented (Covaris M220; Covaris) and transformed to barcoded sequencing libraries using GeneRead DNA Library L Core Kit (Qiagen) according to the manufacturer’s instructions. The resulting libraries were size-selected using AMPureXP beads (Beckmann-Coulter), qualified (Bioanalyzer 2100; Agilent) and quantified with the KAPA Library Quantification Kit Ion Torrent (Roche), and thereafter prepared for equimolar multiplexed sequencing using the Ion 316 Chip Kit v2 on the Ion PGM System (Life Technologies) according to the manufacturer’s instructions. Ion PGM Hi-Q chemistry (Life Technologies) was used for template preparation and sequencing. Raw reads were assembled and thereafter mapped along their respective consensus sequence for variant and SNP calling with 454 Sequencing Systems Software (v3.0; Roche).

Amplicon sequencing and variant calling. For amplicon sequencing, viral RNA was prepared from infected BSR T7/5 cells (MOI 0.01, 3 dpi). To this end, the cells were trypsinized and suspended in 5 mL cell culture medium. After a 5 min centrifugation step at 1500× *g*, the cell pellet was resuspended in 100 µL lysis buffer (10 mM NaCl, 10 mM Tris (pH = 7.5), 1.5 mM MgCl_2_, 1% (*v*/*v*) Triton-X-100, 20 µg micrococcal nuclease (NEB)). After incubation for 30 min at 30 °C, the RNA digest was stopped by the addition of TRIzol^TM^ Reagent and the RNA was prepared. 

By RT-PCR amplification with specific primers carrying a gene-specific and a sequencing platform-specific part (Supplementary Materials, Tables S1 and S2), amplicons were generated. In detail, the amplicons were located in G and P/M: G-1 (308 bp, encompassing aa position 247), G-2 (391 bp, encompassing aa positions 400 and 425), and P/M (280 bp), encompassing aa position 293). For G-1 and G-2, the amplicons were generated to be compatible with Ion Torrent technology using gene-specific primers with full overhang sequencing adapters in a single step. Amplicons were generated for sequencing in forward and backward direction in order to determine potentially present strand bias. Amplicons from different regions and different directions were quantified, pooled equimolarly, and sequenced as described for full length RABV. 

Since in the analyzed P/M region comprises a homopolymer sequence, the amplicons were generated to be compatible with Illumina technology. Here, for library preparation a two-step PCR protocol was employed. First, a set of gene-specific primers with overhang linker adapters were used for RT-PCR followed by a limited-cycle PCR with primers carrying Illumina specific dual index adapters. The amplicons were quantified with the KAPA Library Quantification Kit Illumina (Roche) and sequenced on an Illumina MiSeq instrument in combination with MiSeq reagent Kit v3 (Illumina).

Subsequently, libraries were quality checked and adapter sequences were removed using 454 Sequencing Systems Software (v3.0; Roche) prior to mapping with bowtie2 (v2.4.1) against the reference LN879480. Mapped reads were filtered for the specific gene regions and only reads covering primer to primer were selected for further analysis. A random subset of each 10,000 reads from both mapping directions was selected and analyzed for sub-consensus variants using LoFreq (v 2.1.3.1).

Antibodies, sera, and chromatin stain. The monospecific rabbit sera N161-5 and P160-5 against RABV N and P proteins and the mouse monoclonal antibody E559 against RABV G protein have been described previously [31,32]. Rabbit polyclonal anti-calnexin (1:500 in PBS) and Alexa Fluor-conjugated secondary antibodies (1:1000 in PBS) were obtained from Thermo Scientific. Hoechst33342 (1 µg/mL; Sigma–Aldrich, Burlington, MA, USA) was used for staining of nuclear chromatin.

Indirect immunofluorescence, confocal laser-scanning microscopy, and image processing. For indirect immunofluorescence detection, cells growing on glass cover slips were fixed with 4% paraformaldehyde (PFA) in phosphate buffered saline (PBS). After 30 min incubation at RT, the PFA was removed and the specimens were blocked by incubation with 0.025% skim milk powder in PBS for 20 min at RT. Immunostainings were performed by a two-hour incubation with primary antibody, two wash steps with PBS, and a subsequent 45 min incubation with Alexa Fluor-conjugated secondary antibodies. After two wash steps with PBS, the specimens were mounted on object slides using Kaiser’s glycerol gelatin.

Images were acquired with a Leica DMI6000 TCS SP5 confocal laser scan microscope (63× objective; numerical aperture: 1.4) with sequential acquisition of the fluorophores. Images were processed with the ImageJ software version 1.48b [33].

Electron microscopy. For EM analysis, BSR T7/5 monolayer cells were infected at an MOI of 0.01 and fixed at 72 h post-infection for 120 min with 2.5% glutaraldehyde buffered in 0.1 M Na-cacodylate, pH 7.2, 300 mOsmol (Merck, Darmstadt, Germany). Cell pellets were embedded in low melting agarose (Biozym, Oldendorf, Germany) and cut in small pieces. After that the samples were postfixed in 1.0% aq. OsO_4_ (Polysciences Europe, Eppelheim, Germany) and stained with uranyl acetate (Serva Electrophoresis, Heidelberg, Germany). After stepwise dehydration in ethanol, cells were cleared in propylene oxide, infiltrated with Glycid ether 100 (Serva Electrophoresis, Heidelberg, Germany), and polymerized at 60 °C for 3 days. Ultrathin sections were prepared with an ultramicrotome (UC7 Leica Microsystems, Germany) and collected on 300 mesh EM grids (Nickel, Plano GmbH, Wetzlar, Germany). The grids were counterstained with uranyl acetate and lead citrate, and finally examined with a transmission electron microscope (Tecnai Spirit, Eindhoven, The Netherlands) at an accelerating voltage of 80 kV.

Statistical Analysis. Statistical significance was determined using one-way ANOVA followed by Tukey’s multiple comparison test using GraphPad Prism (v8.1.0).

## 3. Results

### 3.1. Cell Culture Adaptation of rRABV Dog and rRABV Fox

**Figure 1:**
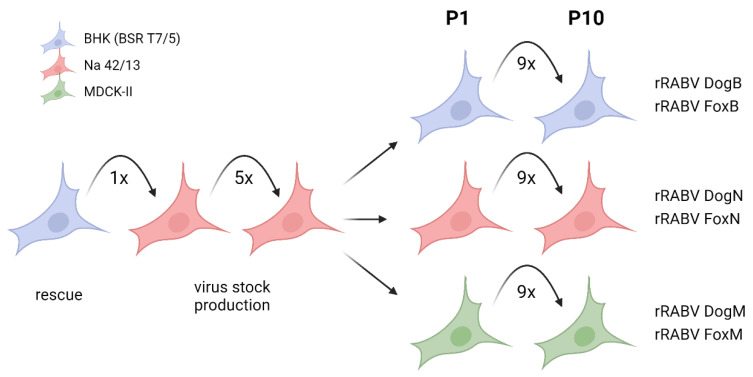
Passage history of the recombinant virus clones rRABV Dog and rRABV Fox. After rescue of the recombinant virus clones rRABV Dog and rRABV Fox in BSR T7/5 cells, five successive supernatant virus passages were performed on Na 42/13 for virus stock preparation. The viruses were then serially passaged ten times (P1–P10) on BHK (BSR T7/5), Na 42/13, or MDCK-II cells. Dependent on the respective cell line, a suffix was added to the resultant viruses’ designation (B/N/M = passage history on BHK/Na 42/13/MDCK-II cells).

Recombinant virus clones rRABV Dog and rRABV Fox were derived from field virus cDNA clones prRABV Dog and prRABV Fox, and serially passaged on murine Na 42/13 neuroblastoma, fibroblastoid baby hamster kidney (BHK-21 clone BSR T7/5), and epithelial Madin–Darby canine kidney cells (MDCK-II) (Figure 1). Prior to the passages on different cell lines, the viruses had been rescued in BSR T7/5 cells [13] and passaged five times on Na 42/13 cells for virus stock preparation.

To assess putative adaptation events in the passaged viruses, supernatants were collected from each passage and the infectious virus titers was determined. Notably, for rRABV DogB-P1 and -P10 an increase of the infectious virus titer from 3 × 10^4^ to 1 × 10^7^ focus forming units (ffu) was observed over time. Also, a 30-fold titer increase was observed for rRABV FoxB-P10 (Figure S1a). These data indicated that passages of rRABV Dog and rRABV Fox on BHK cells might have led to titer-increasing adaptive mutations. On the contrary, passages on Na 42/13 cells did not lead to a substantial titer increase for rRABV DogN-P10 and rRABV FoxN-P10 (Figure S1b). While titers of MDCK-II cell-passaged rRABV DogM-P10 increased by 1.5 log, the high titer for rRABV DogM-P1 (1 × 10^6^ ffu/mL) strongly decreased for the P2 virus and then slowly increased again in later passages (Figure S1c). 

### 3.2. Replication Kinetics of Cell Culture-Passaged rRABV Dog and rRABV Fox

**Figure 2:**
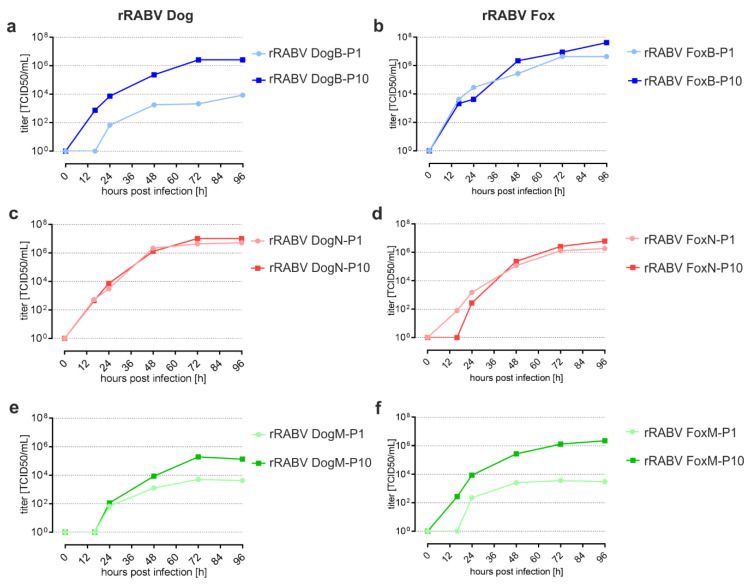
Replication kinetics of cell culture-passaged rRABV Dog and rRABV Fox. Replication curves were determined after infection of the respective cell lines at an MOI of 0.01. (**a**) rRABV DogB-P1 and -P10 on BHK cells. (**b**) rRABV FoxB-P1 and -P10 on BHK cells. (**c**) rRABV DogN-P1 and -P10 on Na 42/13 cells. (**d**) rRABV FoxN-P1 and -P10 on Na 42/13 cells. (**e**) rRABV DogM-P1 and -P10 on MDCK-II cells. (**f**) rRABV FoxM-P1 and -P10 on MDCK-II cells. Shown are representative replication curves for the indicated viruses.

To test whether virus replication in cell culture was indeed affected by the passages on BHK, Na 42/13, and MDCK-II cells, growth curves for the P1 and P10 viruses were performed on the respective cell lines. After infection at an MOI of 0.01, cell culture supernatants were collected for virus titrations at 0, 16, 24, 48, 72, and 96 hpi (hours post-infection). Notably, the rRABV DogB-P10 titers were ~100-fold higher than those of the rRABV DogB-P1 from 16 hpi onwards (Figure 2a), confirming adaptation and improved replication of rRABV DogB-P10 on BHK cells. In contrast to that, the BHK cell passages of rRABV FoxB did not lead to obvious alterations in replication behavior (Figure 2b). Neither did the Na 42/13 passages of rRABV DogN and rRABV FoxN (Figure 2c,d). For rRABV DogM and rRABV FoxM, an increased virus production was suggested by higher virus titers of the P10 viruses, indicating adaptation of these viruses to MDCK-II cells. Overall, these data indicated that adaptation of RABV to respective cell lines had occurred within ten successive virus passages and that the requirement of adaptation might be host cell dependent.

### 3.3. Intracellular Accumulation of rRABV DogB-P1 and rRABV DogM-P1 Viruses in BHK and MDCK-II Cells 

**Figure 3:**
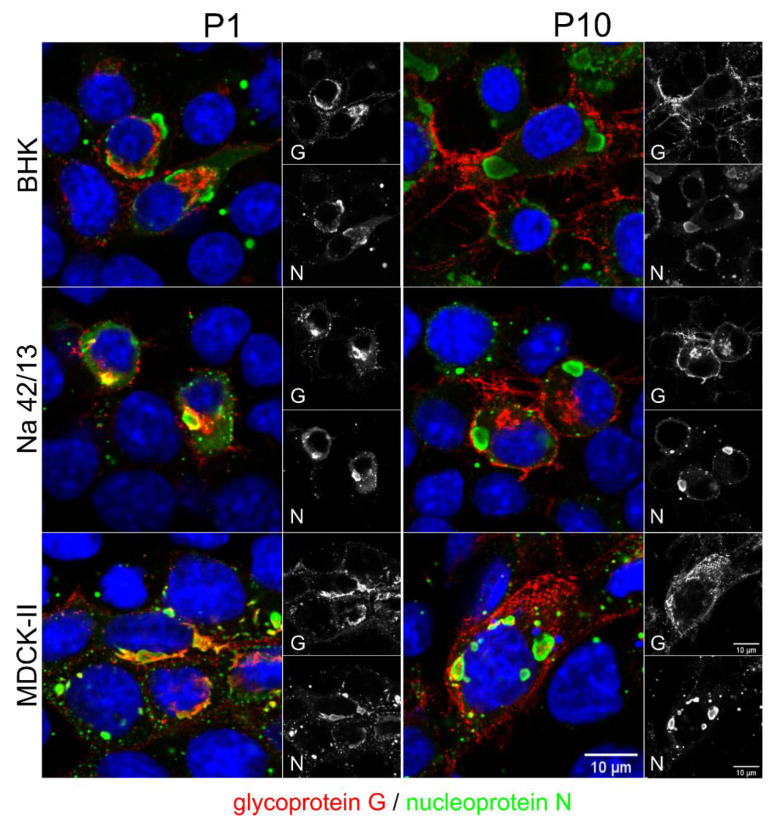
Early passages of rRABV Dog on BHK, Na 42/13, and MDCK-II cells underline an intracellular accumulation of glycoprotein G. Immunostaining of glycoprotein G (red) and nucleoprotein N (green) in cells infected with P1 and P10 viruses at 1 dpi. The distribution of G changes from a perinuclear localization in cells infected with P1 viruses to a more plasma membrane-associated localization in cells infected with P10 viruses. Shown are representative images for the indicated cell lines. Blue: Hoechst 33342.

To assess whether the serial passaging of the viruses led to obvious alterations in the intracellular distribution of virus proteins, immunofluorescence analysis of BHK, Na 42/13, and MDCK-II cells infected with the rRABV DogB/N/M-P1 and -P10 viruses at an MOI of 0.01 were performed (Figure 3). Strong perinuclear accumulation of G was observed for rRABV DogB-P1 and rRABV DogM-P1. Plasma membrane localization of RABV G was barely detectable. In rRABV DogN-P1-infected Na 42/13 cells, G accumulated intracellularly, however, more G signals were also detected in the cell periphery when compared to rRABV DogB-P1 and rRABV DogM-P1.

After the ten virus passages, all viruses exhibited a pronounced plasma membrane localization of the glycoprotein G, while the intracellular G accumulation was strongly reduced. These data indicated that the G protein of the field virus rRABV Dog accumulates within the infected cells and that serial cell culture passages led to an increased cell surface presentation of the viral glycoprotein. 

**Figure 4:**
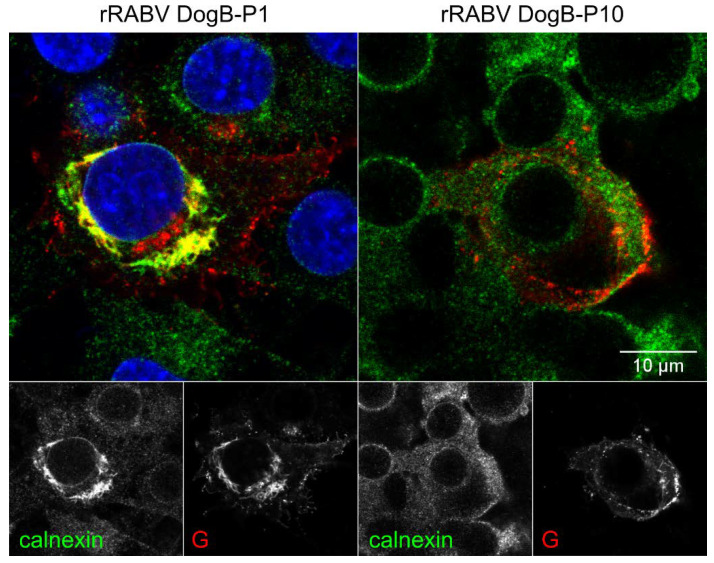
G protein retention in the endoplasmatic reticulum (ER). Immunofluorescence staining of RABV G (red) and the ER marker calnexin (green) in BHK cells at 1 dpi underlines an accumulation of G within the ER for the P1 virus. A loss of ER localization is observed following the serial passaging in rRABV DogB-P10-infected cells. Blue: Hoechst 33342.

Co-localization studies of rRABV DogB-P1 G with the endoplasmatic reticulum (ER) marker calnexin further revealed that the G protein of non-BHK-adapted rRABV DogB-P1 accumulates in the ER (Figure 4). Following the serial passages, less G protein appeared to localize in the ER in rRABV DogB-P10-infected cells. Notably, G accumulation in the ER was associated with a condensation of calnexin signals to perinuclear regions, indicating a structural reorganization of the ER in the course of G protein accumulation.

### 3.4. Deep Sequence Analysis of P10 Viruses

Deep sequence analysis of all six P10 viruses (Figure 1) was performed with RNA isolated from BHK, Na 42/13, and MDCK-II cells infected at an MOI of 0.1 at 2 dpi. The consensus sequences obtained by Ion Torrent sequencing were compared with the parental full-length cDNA clones used for the rescue of recombinant rRABV Dog and rRABV Fox. 

For rRABV DogB-P10, N-P10 and M-P10, four, three, and five amino acid (aa) substitutions were identified, which exceeded a frequency of 50% of reads, respectively (Table 1). All sequenced viruses featured aa substitutions in the glycoprotein. The majority of aa substitutions occurred in the ectodomain of G. In four out of the six P10 viruses, one additional aa substitution was identified in the phosphoprotein, whereas only a single virus exhibited mutations in either M or L. No alterations were observed in the nucleoprotein.

Interestingly, identical aa exchanges in G were identified in both passaged rRABV Dog and rRABV Fox viruses in a host cell-independent and -dependent manner. Of the rRABV DogB-P10 glycoprotein mutations D247N, A400T, and K425N, the D247N exchange was identified in all P10 viruses, whereas K425N specifically only appeared in BHK cell-passaged rRABV DogB-P10 and rRABV FoxB-P10, but not in Na 42/13 and MDCK-II cell-passaged viruses. These data indicated that D247N represents a general adaptive mutation, which independently appeared in the rRABV Dog and rRABV Fox viruses, either in the course of virus stock production on Na 42/13 cells or in subsequent cell passages. In contrast to D247N, independent selection of K425N in BHK cell-passaged rRABV DogB-P10 and rRABV FoxB-P10 without appearance in the other P10 viruses indicated its significance as a host cell-specific adaptation possibly required for increased virus replication in BHK cells. A400T only appeared in rRABV DogB-P10 and thus may represent a rRABV DogB-specific adaptive mutation. Other unique and abundant aa substitutions in G were identified in rRABV DogN-P10 (T187M), rRABV DogM-P10 (R264L and R346S), and rRABV FoxM-P10 (R350G and S379A). Similar to the K425N mutation in BHK cell-passaged viruses, arginine replacements R346S and R350G at positions in close proximity to each other in the glycoproteins of MDCK II-passaged rRABV DogM-P10 and rRABV FoxM-P10 may represent host cell-specific adaptations.

Notably, beyond the G mutations, amino acid exchanges at an identical position in the C-terminal region of the phosphoprotein (R293C and R293H) were identified in two independent viruses, rRABV DogB-P10 and rRABV FoxM-P10, respectively, suggesting additional adaptation within the P gene. Whereas rRABV DogN-P10 and rDogM-P10 also exhibited one aa exchange in P at aa positions 83 and 277, no alterations were observed in the P gene of rRABV FoxB-P10 and rRABV FoxN-P10. 

Overall, the deep sequence analysis highlighted the selection of cell line-dependent and cell line-independent adaptive mutations, which may result in increased cell culture replication of the passaged viruses.

### 3.5. Combined Presence of D247N, A400T, and K425N in rRABV DogB-P10 and Elevated Virus Release Caused by These Mutations

High frequencies of 99.8%, 94.4%, and 99.5% for D247N, A400T, and K425N in rRABV DogB-P10 (Table 1) indicated a relationship between the combined occurrence of these mutations in the G-gene and their shared function. In order to take a closer look at this, cDNA copies of the complete G-coding sequence of rRABV DogB-P10 were cloned in the eukaryotic expression vector pCAGGS. All clones featured the D247N, A400T, and K425N mutations (Table 2). In addition to these mutations, three clones exhibited additional aa replacements (V464F in clones 2 and 5; G156V in clone 6).

**Figure 5:**
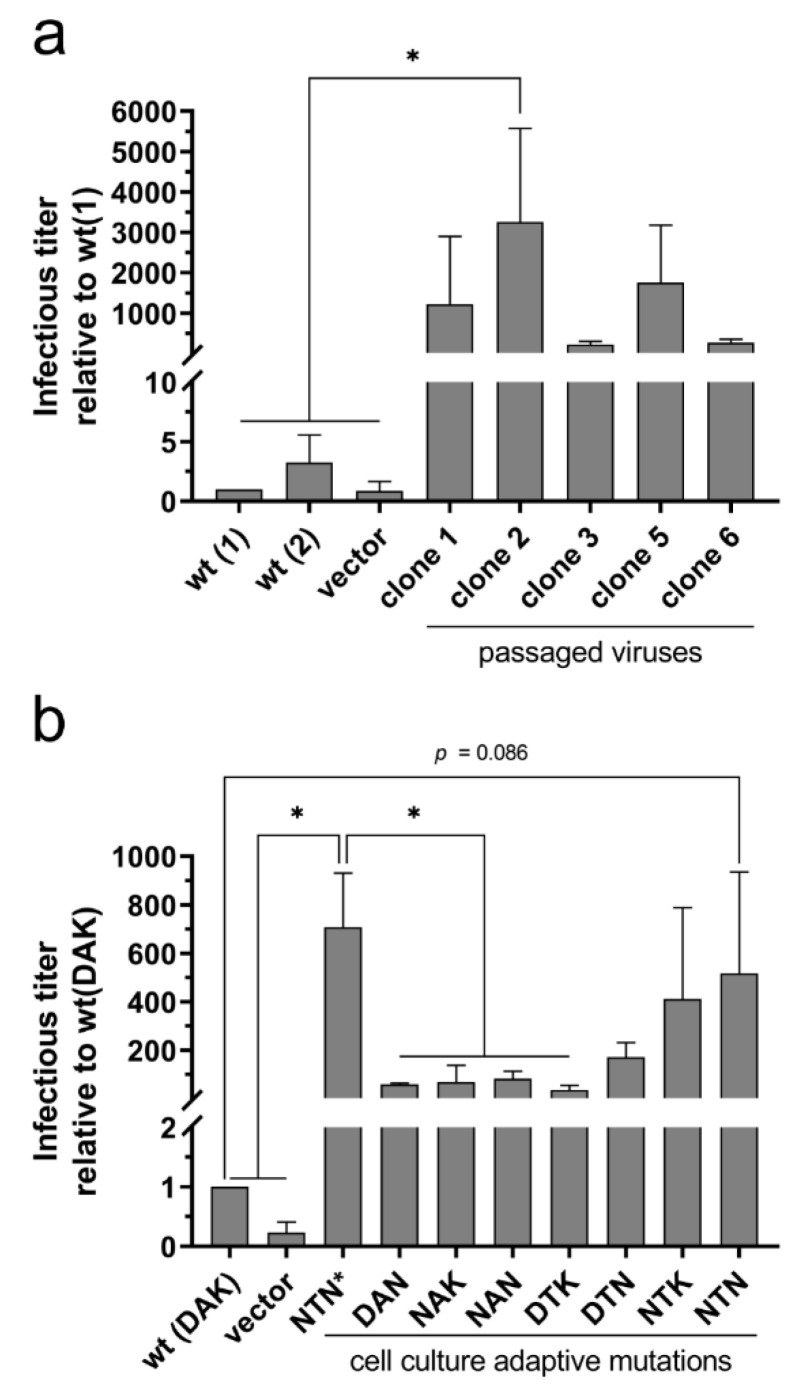
Increased virus release facilitated by the adaptive mutations D247N, A400T and K425N. (**a**) *In trans*-complementation of SAD ΔG-GFP with expression plasmids encoding wild-type rRABV Dog G and five G cDNA clones from rRABV DogB-P10 (clones 1, 2, 3, 5 and 6; Table 2) in BHK cells (clone BSR T7/5). Vector: transfection of the empty vector pCAGGS. Data were normalized to wt rRABV DogA G (wt = 1) (**b**) Complementation of SAD ΔG-GFP with expression plasmids encoding wild-type rRABV Dog G protein (DAK) and G proteins containing different combinations of the adaptive mutations D247N, A400T and K425N. NTN*: full cDNA clone 1 derived from rRABV DogB-P10 (see Figure 5a); NTN: cDNA clone generated by site-directed mutagenesis. Error bars indicate standard deviations from three independent experiments with two independent plasmid clones each (*n* = 6). Statistical significance was determined using a one-way ANOVA followed by Tukey’s multiple comparison test. * *p* ≤ 0.05.

*In trans*-complementation of G gene-deleted and GFP reporter-expressing SAD ΔG GFP rabies virus with clones 1, 2, 3, 5, and 6 revealed that all tested G’s from rRABV-Dog-B-P10 led to increased infectious virus titers when compared to the wild-type dog virus G protein, although a significance level of * *p* ≤ 0.05 was only reached for clone 2 (Figure 5a). These data indicated that virus titers increased with the combinatory presence of D247N, A400T and K425N (clones 1 and 3). An additional positive effect of aa substitutions G157V and V464F (clones 2, 5, and 6) remains to be determined. Western blot detection of the G proteins in transfected BHK cells from complementation assays indicated that the increased virus titers of P10 G-complemented viruses were not due to an overall increase in G protein levels when compared to the wild-type G protein level (Figure S2).

To explore whether a combination of all three mutations in rRABV DogB-P10 indeed was required for increased virus titers on BHK cells, complementation experiments were performed with G variants comprising the individual mutations D247N (NAK), A400T (DTK), and K425N (DAN) as well as different combinations thereof (Figure 5b). Whereas the combinations DAN, NAK, NAN, DTK, and DTN increased the virus titers 59, 70, 84, 36, and 172-fold, respectively, a 412-fold increase was determined for NTK (D247N and A400T). NTN* (cDNA clone 1, Table 2) and NTN (mutant generated by site-directed mutagenesis), both containing D247N, A400T, and K425N, increased the titers 708- and 518-fold, respectively. These data showed that the single aa replacements (DAN, NAK, DTK) already led to increased infectious virus release. Combinations of D247N and A400T (NTK) as well as D247N, A400T, and K425N (NTN) were necessary to achieve an additional increase in infectious virus release.

### 3.6. Acquisition of Adaptive Mutations in rRABV Dog over Time

To investigate the time course over which the mutation accumulated, genome regions comprising the phosphoprotein mutation R293C (382 kb cDNA fragment P/M), glycoprotein mutation D247N (308 bp cDNA fragment G-1), and A400T/K425N (391 kb cDNA fragment G-2) were amplified by RT-PCR from rRABV DogB virus passages P1, P3, P5, P7, and P10, and amplicon sequencing was performed.

The amino acid exchange R293C in P (Table 1, DogB) was first identified after five passages at a frequency of 15%, which then increased to 75% and 99% in passages seven and ten, respectively. Two additional mutations in P were detected at low but stable frequencies, one with an amino acid exchange (L276M; 2% at all time points) and one silent mutation at nucleotide (nt) position 2338 (10% to 12% at all time points). 

In G, the amino acid exchange at position 247 (D247N) was already present in P1, as indicated by 99.8% frequency after the first passage. In amplicon G-1, additional non-silent single-nucleotide polymorphisms (SNPs) leading to amino acid substitutions S165P and T187M were detected (2% and 7.3% in G, respectively), however, were not detectable anymore at P7 and P10. 

In the G-2 amplicon, both A400T and K425N were not detectable in P1 to P3. K425N was first detected in P5 at a frequency of 75% and then increased to 96% and 100% in P7 and P10, respectively. A400T first appeared in P7 at a frequency of 58% and rose to 96% in P10. An additional mutation at position 428 (S428L) was observed at low frequencies (8% to 1%) in P3 and P7, and a synonymous SNP appeared at 99–100% at nt position 4725 already in P1.

In summary, these data revealed that D247N was already present in the rRABV DogB-P1 and that K425N was acquired between passages P3 and P5. A400T was acquired at a later time point between passages P5 and P7. Notably, once the mutations appeared, only few virus passages were required to replace almost the complete original sequence (see frequencies of 96–100% in Table 3 for R293C in P and A400T/K425N in G).

### 3.7. Intracellular Accumulation of rRABV Dog G Protein in BHK Cells and Increased G Cell Surface Localization by D247N, A400T, and K425N

Since rRABV DogB-P1 already contained the D247N mutation (Table 3), a new recombinant virus was rescued from cDNA in Na 42/13 cells, in which the absence of any of the adaptive mutations in G after three passages on Na 42/13 cells was confirmed by RT-PCR product sequencing of the stock virus. The resultant virus was dubbed rRABV Dog*. In addition, the recombinant dog virus mutant rRABV DogB-NTN was generated by insertion of the three adaptive mutations D247N, A400T, and K425N in the RABV Dog cDNA full length clone.

**Figure 6:**
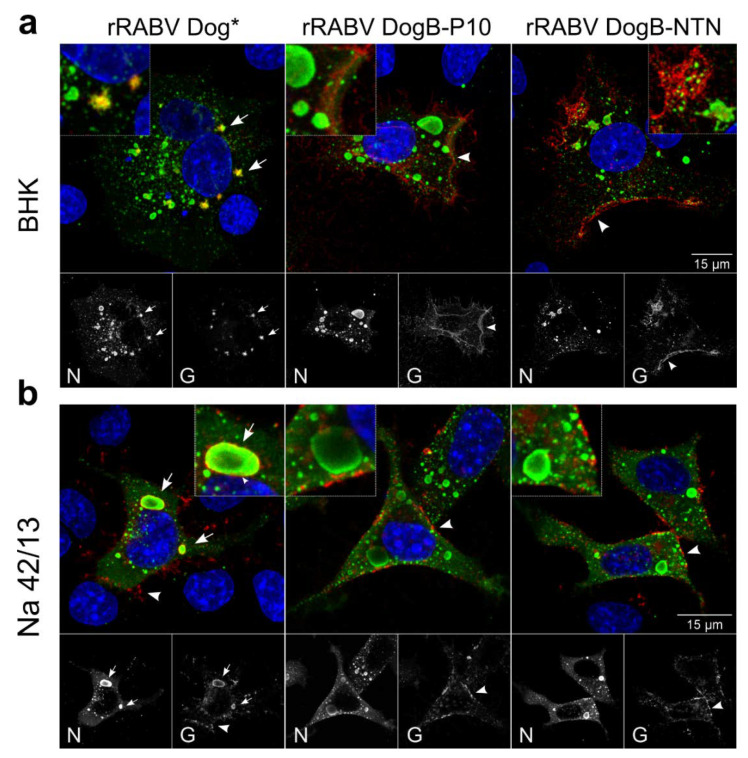
Increased cell surface accumulation of rRABV DogB-P10 and rRABV Dog-NTN G proteins. rRABV Dog*, rRABV DogB-P10, and rRABV Dog-NTN infection of BHK (**a**) and Na 42/13 (**b**) cells at 1 dpi. Confocal laser-scanning analysis was performed after immunostaining for glycoprotein G (red) and phosphoprotein P (green). Blue: Hoechst 33342. Arrows: inclusion body-associated G; arrowheads: plasma membrane localization of G.

After infection of BHK cells with rRABV Dog*, intracellular G protein accumulations were detected at cytoplasmic inclusion bodies (Figure 6a, arrows), whereas G accumulations at the plasma membrane were not visible. In striking contrasts, both rRABV DogB-P10 and rRABV DogB-NTN glycoproteins were not detected at cytoplasmic inclusion bodies but accumulated at the plasma membrane of infected cells (arrowheads). These data showed that the cellular localization of the RABV Dog G protein was affected by the introduced mutations and that accumulation of abundant G protein levels at the plasma membrane correlated with the presence of the three adaptive mutations D247N, A400T, and K425N.

In Na 42/13 cells, rRABV Dog* also led to inclusion body-associated G accumulation (Figure 6b; arrows), however, G was also detected at the plasma membrane (arrowheads), indicating lower necessity for adaptation in Na 42/13 than in BHK cells. The loss of a pronounced inclusion body association in rRABV DogB-P10- and rRABV DogB-NTN-infected cells together with accumulation of mutated G in the cell periphery again demonstrated an NTN-dependent increase of plasma membrane accumulation. 

### 3.8. Accumulation of Virus Particles at the Cytoplasmic Inclusion Bodies

**Figure 7:**
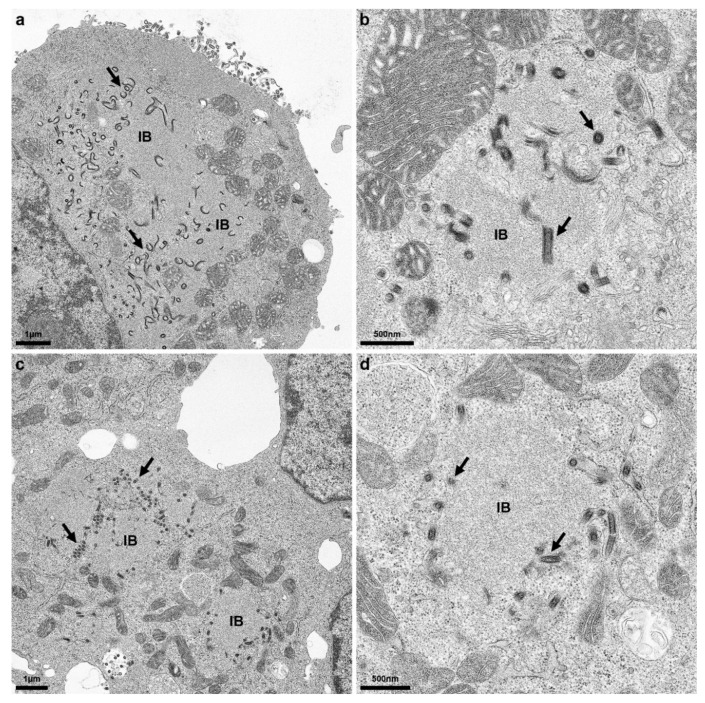
Accumulation of intracellular virus particles at inclusion bodies in rRABV Dog-infected BHK cells. Na 42/13 (**a**,**b**) and BHK cells (**c**,**d**) were infected at an MOI of 0.01. Three dpi, cells were fixed and analyzed by transmission electron microscopy. Note: Electron microscopy analysis was performed with rRABV Dog already comprising the D247N mutation. Arrows: accumulation of intracellular virus particle at the surface of cytoplasmic inclusion bodies.

Electron microscopy analysis of rRABV Dog infected Na 42/13 and BHK cells confirmed intracellular accumulation of virus particle structures at the interface of the rough endoplasmic reticulum (rER) and inclusion bodies (Figure 7). Whereas infected Na 42/13 cells revealed more budding at the plasma membrane, less extracellular virus particles were observed at the plasma membrane of BHK cells (not shown). Virus particle accumulations were observed at the surface of cytoplasmic inclusion bodies (Figure 7, arrows).

## 4. Discussion

By serial passaging of two recombinant field virus RABV clones of highly virulent fox and dog isolates [13], we identified cell culture-adaptive amino acid exchanges, which were mainly located in the glycoprotein G, and, to a minor extent, in the C-terminal part of phosphoprotein P (Table 1). Notably, the appearance of adaptive mutations in the G protein was highly reproducible and identical amino acid replacements were independently selected by the two different RABV field virus clones of dog and fox virus isolate origin. Moreover, cell line-specific mutations in both viruses showed that cell-specific constraints must be overcome to support efficient RABV amplification in these particular cell lines.

Similar to previous studies, where an increased glycosylation status of G was achieved by the D247N mutation, which was the result of neuroblastoma cell culture adaptation of the field virus strain 1088 [19,21], here we identified the respective D247N mutations in both cell culture-passaged rRABV Dog and rRABV Fox. As shown for the RABV strain 1088 G protein by *in trans*-complementation assays with G gene-deleted RABV [20], our complementation assays with mutated dog virus G variants also indicated an improvement of virus release caused by the D247N mutation alone (Figure 5). However, additional mutations were required for a significant increase of infectious virus titers in cell culture supernatants. The presence of the D247N mutation already in the P1 virus after five passages on Na 42/13 (Table 3) together with the lack of detectable titer increases after ten passages on Na 42/13 cells (Figure 2c,d) strongly indicate that D247N alone was sufficient to mediate efficient replication in Na 42/13 cells. However, we cannot exclude that the additional T187M mutation was also required for a more efficient replication of rRABV Dog on Na 42/13 cells, as it reached a frequency of 97.9% in rRABV DogN-P10 (Table 1) and increased the cell surface accumulation of G (Figure 3). D247N and T187M are located in the pleckstrin homology domain (PHD), and in the linker connection between the PHD and the fusion domain, respectively, according to the structure by Yang et al. [6]. Consequently, these two mutations could be structurally linked and may facilitate G transport to the cell surface. 

We also cannot exclude minor effects of the adaptive mutations on the susceptibility of the cells to virus infection. However, observed phenotypes shown for the G distribution shown in Figure 3, Figure 4 and Figure 6, together with the increased infectious virus titers of complemented viruses on BHK cells (Figure 5), strongly indicate that virus release instead of susceptibility is the determining factor in the cell line adaptation described here.

The selection of the D247N mutation in the two cell culture-passaged field viruses here and identification of the same mutation in passaged 1088 virus, SAD B19, RV-97, Nishigahara, and RC-HL vaccine strains [21] further corroborates a highly conserved mechanism of cell culture adaptation by insertion of an additional N-glycosylation site at position 247. However, alternate adaptive glycosylations at positions 158 in the LEP-Flury and 204 in CVS11, CVS-N2c, CVS-26, PM1503, and Kyoto strains [34] indicate RABV strain-specific flexibility in the position of the additional glycosylation site. Taken together, our and previous studies strongly argue in favor of a model, in which infectious virus release is limited by the number of N-glycosylation sites in the G protein’s ectodomain, which thus may represent a bottleneck for cell culture adaptation of field RABVs. Mechanistically, this may rely on structural constraints within the G proteins as glycosylations at positions 247 and 319 are important for correct folding of nascent G and subsequent transport processes in the cell [19,34,35]. Moreover, deletion of all N-glycosylation sites in the rabies virus glycoprotein blocks cell surface accumulation of the G protein altogether [35,36], perhaps by improper protein folding and sequestration in the ER.

Whereas D247N and other mutations lead to NxT/S glycosylation sequons in several RABV strains [20,21,34,35,37], the 425N-426Q-427V sequence in the K425N mutants rRABV DogB-P10 and rRABV FoxB-P10 did not represent an additional potential N-glycosylation site. We assume that other glycosylation-independent modifications led to the observed improvement in virus replication and release in BHK cells. Comparable to K425N in rRABV DogB-P10 and rRABV FoxB-P10, the R346S and R350G mutations in MDCK-II cell-passaged viruses did not generate potential glycosylation sequons. Therefore, we also assume that additional glycosylation-independent adaptation was required in MDCK-II cells. Considering the occurrence of the K425N mutation in two different BHK cell-passaged field virus clones (rRABV DogB-P10 and rRABV FoxB-P10) and the identification of potential MDCK-II-specific mutations (R346S in rRABV DogM-P10 and R350G in rRABV FoxM-P10; Figure S3), a cell line-specific selection of adaptive mutations is evident. Accordingly, these data indicate that, in addition to increased N-glycosylation, cell culture adaption requires additional glycosylation-independent mutations, which can be cell line-specific. Both the BHK-specific and the potentially MDCK-II-specific mutations were located in the central domain (CD). Localization of R346S and R350G in short two-amino-acid beta sheets may affect structural refinement within the CD. In contrast, the BHK-specific mutations A400T and K425N were located either at the extended C-terminal tail of the CD or in case of K425N outside of the resolved crystal structure [6].

In BHK cells, the combination of D247N, A400T, and K425N overcame either cell-specific intracellular retention or rapid re-internalization of non-adapted glycoprotein G, as demonstrated by the intracellular accumulation of non-mutated G protein at cytoplasmic inclusion bodies of rRABV Dog* and increased plasma membrane accumulation of G protein in rRABV DogB-NTN-/rRABV DogB-P10-infected BHK cells (Figure 6A). rRABV Dog* was newly generated from the respective full-length plasmid clone directly in Na 42/13 cells. For propagation, it was passaged only three times on Na 42/13 cells and the D247N mutation was excluded by Sanger sequencing of the virus stock. Strong, almost exclusive accumulation of G at cytoplasmic inclusion bodies in BHK cells and a clear inclusion body-associated localization in Na 42/13 cells confirmed the intracellular retention of the field virus G and further revealed that, without adaptative mutations, the G protein accumulated in close proximity to the inclusion bodies (Figure 6). Whether the comparable effect of the NTN mutations in Na 42/13 cells (Figure 6B) was due to the combination of the three mutations or whether the D247N alone was able to increase plasma membrane accumulation of the glycoprotein has not been further investigated.

Rabies virus cytoplasmic inclusion bodies (IBs) are liquid organelles and sites of virus RNA synthesis [38,39], which comprise the viral N, P, M, and L proteins [40]. G protein is not considered to accumulate in IBs. However, intracellular accumulation of enveloped rabies virus particles in cisterna of degranulated and dilated rER is well documented. In cell cultures, these intracellular virus accumulations are considered not to contribute to infectious virus release and the role in the rabies virus life cycle remains unsolved thus far [27,40]. Intracellular IB-associated G accumulations in rRABV Dog* may represent increased virus particle budding in the rER at the interface of IBs and the intracellular membrane compartment, which may also result in reorganization of the rER (Figure 3). This is also supported by the identification of enveloped virus particles in close vicinity to viral inclusion bodies (Figure 7). Accordingly, G surface transport in rRABV DogB-P10- and rRABV Dog-NTN-infected cells may represent a shift in the balance of intracellular and plasma membrane budding, of which the latter is decisive for infectious virus titers in the cell culture supernatants. Successive supernatant passages over the course of cell culture adaptation thus represent the major driving force for the selection of virus mutants shifted towards increased plasma membrane budding. 

Although a combination of all three G mutations (D247N, A400T, K425N) was identified in rRABV-DogB-P10, stepwise selection of K425N and A400T indicated a possible requirement of K425N for rapid selection of A400T (from 0% at P5 to 59% at P7 and to 97% at P10; Table 3). The requirement of A400T for a significant titer increase in complementation experiments in combination with K425N (Figure 5B) corroborated this interpretation, although A400T seemed to have a positive effect on virus release even in combination with D247N alone (Figure 5B).

Notably, a minor mutation (T187M) present at a frequency of 8.5% in rRABV DogB-P1 was lost in BHK-passaged rRABV DogB-P10, while it was established as a major amino acid replacement in neuroblastoma cell-passaged rRABV DogN-P10, indicating a positive selection of this mutation on Na 42/13 cells. In contrast to K425N, which appeared in both rRABV DogB-P10 and rRABV FoxB-P10, this mutation was not identified in rRABV FoxN-P10. Although, the wild-type G protein ectodomain sequences of the rRABV Fox and Dog field viruses differ at only three positions (179L, 236I, and 353H in rRABV Fox, and 179M, 236M, and 353P in rRABV Dog; Figure S3), it is conceivable that one or a combination of these substitutions made the accumulation of mutations such as T187M redundant in the context of rRABV Fox. This could be due to the virus isolate-specific component in cell culture adaptation. 

It is also conceivable, that the 179L, 236I, and 353H residues in rRABV Fox already led to a certain degree of adaptation in BHK cells, as indicated by Figure 2b. However, the K425N exchange in the glycoprotein G has also been selected for in the P10 virus of rRABV FoxB (Table 3), strongly indicating that K425N was also supportive for virus replication in BHK cells. Although presence of K425N in the consensus sequence was excluded by Sanger sequencing of pre-passage rRABV Fox (not shown), we cannot exclude that a minor fraction of rRABV Fox-P1 already contained the K425N, potentially acquired early in the course of virus rescue on BHK cells, which may lead to rapid adaptation already in the rRABV Fox-P1.

According to its host cell-specific character, the K425N mutation was not detected after initial amplification on Na 42/13 cells (Figure 1, Table 1), but after three passages on BHK cells (Table 3). Other RABVs with documented BHK cell passage history have accumulated the K425N substitution in the glycoprotein G as well, including an RABV isolate from a ferret-badger [41]. Notably, also several SAD vaccine virus variants contain the K425N mutation [41]. Combined presence of 187M, 247N, and 425N in SAD L16 virus (Figure S3) derived from the vaccine strain SAD B19 [24] underlined the combinatory character of the adaptive mutations in the rRABV Dog and rRABV Fox G proteins selected here. The stability of 187M in the presence of 247N and 425K in SAD L16 G suggests that one or more of the other 15 amino acid differences to the glycoproteins of rRABV Dog and rRABV Fox may have stabilized this combination over multiple cell passages. Taken together, we were able to generate mutated field viruses, which exhibited an amino acid signature comparable to a BHK cell-passaged vaccine virus by five passages on Na 42/13 cells (virus stock preparation) and ten additional passages on BHK cells. Whether these adaptive mutations affect virulence and pathogenicity in vivo has to be investigated. Indeed, additional glycosylation and further mutations in G correlate with reduced pathogenicity and stronger immune responses in mice [18,21]. Future pathogenicity studies with the recombinant field virus clones used here may allow comparative analyses in vivo to clarify whether increased virus release from infected cells is advantageous or disadvantageous for RABV pathogenesis in the host.

As outlined earlier, site-directed mutagenesis and rescue of the recombinant rRABV DogB-NTN, comprising D247N, A400T, and K425N, allowed confirmation of mutation-dependent loss of G protein accumulation at intracellular viral inclusion bodies (Figure 6) and increased surface accumulation at the plasma membrane. Together with *in trans*-complementation of G gene-deleted SAD ΔG virus (Figure 5), these data show that a 100-fold titer increase exclusively depends on the presence of these three mutations and that a major bottleneck in cell culture replication of field RABVs is low level virus release because of limited cell surface accumulation of G. However, these data did not exclude further beneficial effects of additional mutations outside the G gene identified in coding and non-coding sequences of the P10 viruses.

While individual mutation events in P, M, and L (P-M83R, P-L277V, M-D38Y, and L-A1535S) may represent variations by chance, two amino acid exchanges in the C-terminal region of the phosphoprotein P were selected in two independent viruses on two different cell lines with P-R293C and P-R293H in rRABV DogB-P10 and rRABV FoxM-P10, respectively. Considering the low overall frequency of non-silent mutations observed (Table 1), these data strongly indicated selection pressure for the replacement of the arginine residue at that position, although it only appeared in two out of six passaging approaches. Since RABV P is a polymerase cofactor, chaperons the nucleoprotein N [2], and acts as the major type I interferon antagonist of RABVs [42], multiple effects of the C-terminal mutation are conceivable. The P protein’s C-terminus contains a positively-charged patch and a hydrophobic pocket with an exposed tryptophan side chain at opposite surfaces of the molecule [43]. Those two interfaces represent functionally and spatially independent regions with critical roles in genome replication and STAT-binding/antagonism, respectively [44]. Together with lysine residues at positions 211, 214, 256, 260, and 282, R293 forms the positively charged C-terminal patch responsible for nucleoprotein-RNA (N-RNA) complex binding [43]. Although further functional analyzes have to be conducted, we hypothesize that the mutations R293C and R293H in rRABV FoxM-P10 and rRABV DogB-P10 identified here may influence N-RNA binding and virus genome replication. Positive selection of these mutations in two out of six independent virus passaging experiments strongly indicates a positive effect on virus replication in these cell cultures. However, these mutations do not seem to be essential for cell culture adaptation, since no P mutations were identified in rRABV FoxB-P10 and rRABV FoxN-P10. Whether alternative mutations in the C-terminus (L277V) or more upstream (M83R) of the P protein were compensatory to R293C and R293H has to be analyzed in future studies.

## 5. Conclusions

Our data suggest that improved virus release caused by only a few amino acid replacements in the ectodomain of G represents a major difference between cell culture-adapted and non-fixed field RABVs. This may not only be of interest for a better understanding of requirements for RABV cell culture adaptation, but also offers new possibilities to further investigate the impact of defined cell culture-adaptive mutations on virulence and pathogenicity in vivo. Since most fixed laboratory-adapted strains have acquired multiple mutations, with the progenitor isolates often not being available, the recombinant field virus clones with a defined set of cell line-specific adaptive mutations described here offer the possibility to address the question whether and to what extent improved virus release after cell culture adaptation influences RABV infection in the natural host. Whereas virus spread and disease development may possibly be sped up by the more efficient virus release, higher levels of antigen presentation and resultant stronger immune responses could result in an inverse correlation between virus release and virulence. The recombinant field viruses described here now allow the experimental dissection of adaptation, pathogenicity, and attenuation.

## Figures and Tables

**Table 1:** Amino acid exchanges in P10 viruses of rRABV Dog and rRABV Fox. Indicated are the number of silent mutations in the whole genome and amino acid exchanges in the virus proteins N, P, M, G, and L at frequencies higher than 50% of sequence reads in comparison to the consensus sequences of rRABV Dog and rRABV Fox. NCR = non-coding region (excluding transcription signals). Percentages indicate the frequency of sequence reads containing the indicated mutation. Amino acid positions in G refer to the processed glycoprotein without the N-terminal 19 aa signal peptide.

	Mutations in NCR	Synonymous Mutations	Non-Synonymous Mutations				
			N	P	M	G	L
DogB (BHK)	0	1	-	R293C ^iv^ (96.4%)	-	D247N ^i^ (99.8%) A400T (94.4%) K425N ^ii^ (99.5%)	-
DogN (Na 42/13)	2	1	-	M83R (97.5%)	-	T187M (97.9%) D247N ^i^ (100%)	-
DogM (MDCK-II)	0	3	-	L277V (68%)	-	D247N ^i^ (100%) R264L (92.5%) R346S ^iii^ (70.5%)	A1535S (74.7%)
FoxB (BHK)	0	1	-	-	-	D247N ^i^ (99.5%) K425N ^ii^ (76.7%)	-
FoxN (Na 42/13)	0	1	-	-	-	D247N ^i^ (99.5%)	-
FoxM (MDCK-II)	1	3	-	R293H ^iv^ (92.4%)	D38Y (81.6%)	D247N ^i^ (100%) R350G ^iii^ (88.1%) S379A (95.5%)	-

^i^: mutation in all sequenced P10 viruses. ^ii^: BHK cell-specific mutation in rRABV DogB-P10 and rRABV FoxB-P10. ^iii^: mutation in rRABV DogM-P10 and rRABV FoxM-P10 presumably representing MDCK-II cell-specific adaptations. ^iv^: mutation in rRABV DogB-P10 and rRABV FoxM-P10 at identical position in C-terminus of P.

**Table 2:** Combination of mutations in G gene cDNAs cloned from rRABV DogB-P10. Indicated are aa substitutions with respect to the original rRABV Dog sequence.

	*Amino Acid Differences to rRABV Dog*				
clone 1 *	-	**D247N**	**A400T**	K425N	-
clone 2 *	-	D247N	A400T	K425N	V464F
clone 3 *	-	D247N	A400T	K425N	-
clone 4	-	D247N	A400T	K425N	-
clone 5 *	-	D247N	A400T	K425N	V464F
clone 6 *	G156V	D247N	A400T	K425N	-
clone 7	-	D247N	A400T	K425N	-

^*^ clones selected for functional analysis by *in trans*-complementation of SAD ΔG GFP.

**Table 3:** Single nucleotide variants detected by amplicon sequencing of rRABV DogB-P1after 1, 3, 5, 7, and 10 passages on BHK cells. The frequencies of the identified variants are indicated for every passage number. nd: not detected (variant below 1% cutoff). ns: not sequenced. nt: nucleotide position in reference LN879480. aa: amino acid position in P or G (minus 19 aa signal peptide).

	P			G-1			G-2			
nt	2338 (A→G)	2339 (T→A)	2390 (C→T)	3865 (T→C)	3932 (C→T)	4111 (G→A)	4570 (G→A)	4647 (G→T)	4655 (C→T)	4725 (G→A)
aa	none	L276M	R293C	S165P	T187M	D247N	A400T	K425N	S428L	none
P1	10%	2%	nd	2%	7.3%	98%	nd	0%	0%	100%
P3	10%	2%	nd	1%	13%	99%	nd	24%	3%	100%
P5	11%	2%	15%	1%	18%	99%	nd	75%	ns	100%
P7	12%	2%	75%	nd	1%	99%	58%	96%	1%	100%
P10	11%	2%	99%	nd	nd	99%	96%	100%	nd	100%

## Data Availability

Please refer to suggested Data Availability Statements in section “MDPI Research Data Policies” at https://www.mdpi.com/ethics (accessed on 6 August 2021).

## Supplementary Materials

The following are available online at www.mdpi.com/article/10.3390/v13101989/s1, Figure S1: Infectious virus titers in the cell culture supernatant determined over the course of virus passaging on BHK (rRABV DogB), NA 42/13 (rRABV DogN) and MDCK-II (rRABV DogM) cells. Figure S2: Western blot detection of G and N protein in BHK cell extracts from complementation experiments. Figure S3: Comparison of the amino acid sequences of the rRABV Dog, rRABV Fox, and SAD L16 glycoprotein ectodomains. Table S1: PCR primers used for amplicon sequencing of P/M region., Table S2: PCR primers used for amplicon sequencing of G-1 and G-2 region.
