# Supplementary material for: Point Mutations in the Glycoprotein Ectodomain of Field Rabies Viruses Mediate Cell Culture Adaptation through Improved Virus Release in a Host Cell Dependent and Independent Manner
Source: Viruses. 2021 Oct 3;13(10):1989. doi: 10.3390/v13101989 (PMC8538267; doi:10.3390/v13101989)
Supplement: Supplementary file 1 [file viruses-13-01989-s001.zip › Nitschel_et_al_Supplementary Material_final.pdf]

Supplemental Information for:

Nitschel *et al.*

"Point Mutations in the Glycoprotein Ectodomain of Field Rabies Viruses Mediate Cell Culture Adaptation Through Improved Virus Release in a Host Cell-Dependent and -Independent Manner."

Table S1: PCR primers used for amplicon sequencing of P/M region. The oligonucleotides used for the first round of PCR contain a gene-specific sequence region and an instrument-specific linker that is used as target for the sequencing platform specific second PCR.

**1st PCR: Gene-specific primers** (instrument specific linker **/ gene-specific**)	
Illumina_PM_fw	5'-CTTTCCCTACACGACGCTCTTCCGATCT**GGCCAATTCCAAGAAATTCCA**-3'
Illumina_PM_rv	5'-GGAGTTCAGACGTGTGCTCTTCCGATC**GGAAGCCACAGGTCATCGT**-3'
**2nd PCR: Indexing primers** (index **/ binding site to instrument specific linker**)	
Dual Indexed Adapter 97 - P5	5'-AATGATACGGCGACCACCGAGATCTACACGACCTGTAACACT**CTTTCCCTACACGACGCTCTTCCGATCT**-3'
Dual Indexed Adapter 97 - P7	5'-CAAGCAGAAGACGGCATACGAGATTATGCTGGGTGACT**GGAGTTCAGACGTGTGCTCTTCCGATC**-3'
Dual Indexed Adapter 110 - P5	5'-AATGATACGGCGACCACCGAGATCTACACATGTAACTACACT**CTTTCCCTACACGACGCTCTTCCGATCT**-3'
Dual Indexed Adapter 110 - P7	5'-CAAGCAGAAGACGGCATACGAGATATAAGCACGTGACT**GGAGTTCAGACGTGTGCTCTTCCGATC**-3'
Dual Indexed Adapter 123 - P5	5'-AATGATACGGCGACCACCGAGATCTACACGTTTCAGAACACT**CTTTCCCTACACGACGCTCTTCCGATCT**-3'
Dual Indexed Adapter 123 - P7	5'-CAAGCAGAAGACGGCATACGAGATGAGACTGTGTGACT**GGAGTTCAGACGTGTGCTCTTCCGATC**-3'
Dual Indexed Adapter 136 - P5	5'-AATGATACGGCGACCACCGAGATCTACACCACAGGATACACT**CTTTCCCTACACGACGCTCTTCCGATCT**-3'
Dual Indexed Adapter 136 - P7	5'-CAAGCAGAAGACGGCATACGAGATCTATGAAAGTGACT**GGAGTTCAGACGTGTGCTCTTCCGATC**-3'
Dual Indexed Adapter 162 - P5	5'-AATGATACGGCGACCACCGAGATCTACACCGGTGGTAACACT**CTTTCCCTACACGACGCTCTTCCGATCT**-3'
Dual Indexed Adapter 162 - P7	5'-CAAGCAGAAGACGGCATACGAGATTTTGCATTGTGACT**GGAGTTCAGACGTGTGCTCTTCCGATC**-3'

Table S2: PCR primers used for amplicon sequencing of G-1 and G-2 region. The oligonucleotides contain a gene-specific sequence region and an instrument-specific sequence.

**Gene-specific primers** (instrument specific **/ gene-specific**)	
IonTorrent_G1_P1_fw	5'-CCTCTCTATGGGCAGTCGGTGAT**TGGTGGAAAGTGCTCAGGAA-3'**
IonTorrent_G1_P1_rv	5'-CCTCTCTATGGGCAGTCGGTGAT**CCAACTGATCAGGAGGGCA-3'**
IonTorrent_G1_A_IX44_fw	5'-CCATCTCATCCCTGCGTGTCTCCGACTCAGTTGGAGGCCAGCGAT**TGGTGGAAAGTGCTCAGGAA-3'**
IonTorrent_G1_A_IX44_rv	5'-CCATCTCATCCCTGCGTGTCTCCGACTCAGTTGGAGGCCAGCGAT**CCAACTGATCAGGAGGGCA-3'**
IonTorrent_G1_A_IX45_fw	5'-CCATCTCATCCCTGCGTGTCTCCGACTCAGTGGAGCTTCCTCGAT**TGGTGGAAAGTGCTCAGGAA-3'**
IonTorrent_G1_A_IX45_rv	5'-CCATCTCATCCCTGCGTGTCTCCGACTCAGTGGAGCTTCCTCGAT**CCAACTGATCAGGAGGGCA-3'**
IonTorrent_G2_P1_fw	5'-CCTCTCTATGGGCAGTCGGTGAT**CCCTCCTCCAGCAACATATG-3'**
IonTorrent_G2_P1_rv	5'-CCTCTCTATGGGCAGTCGGTGAT**ACCAGCCTTCACAGTCTAGT-3'**
IonTorrent_G2_A_IX44_fw	5'-CCATCTCATCCCTGCGTGTCTCCGACTCAGTTGGAGGCCAGCGAT**CCCTCCTCCAGCAACATATG-3'**
IonTorrent_G2_A_IX44_rv	5'-CCATCTCATCCCTGCGTGTCTCCGACTCAGTTGGAGGCCAGCGAT**ACCAGCCTTCACAGTCTAGT-3'**
IonTorrent_G2_A_IX45_fw	5'-CCATCTCATCCCTGCGTGTCTCCGACTCAGTGGAGCTTCCTCGAT**CCCTCCTCCAGCAACATATG-3'**
IonTorrent_G2_A_IX45_rv	5'-CCATCTCATCCCTGCGTGTCTCCGACTCAGTGGAGCTTCCTCGAT**ACCAGCCTTCACAGTCTAGT-3'**

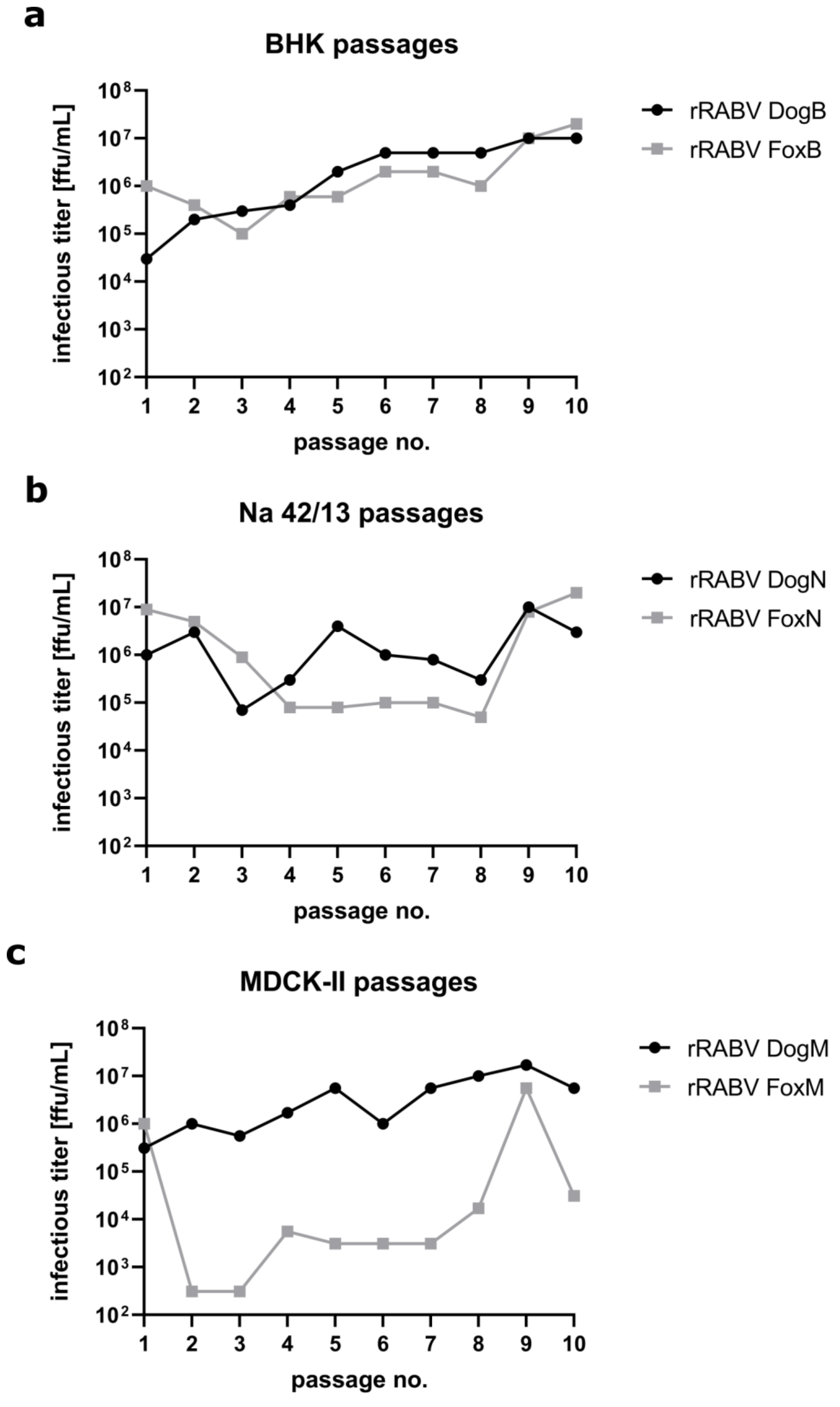

Figure S1: Infectious virus titers in the cell culture supernatant determined over the course of virus passaging on BHK (rRABV DogB), NA 42/13 (rRABV DogN) and MDCK-II (rRABV DogM) cells. Passage numbers are indicated by the x-axis.

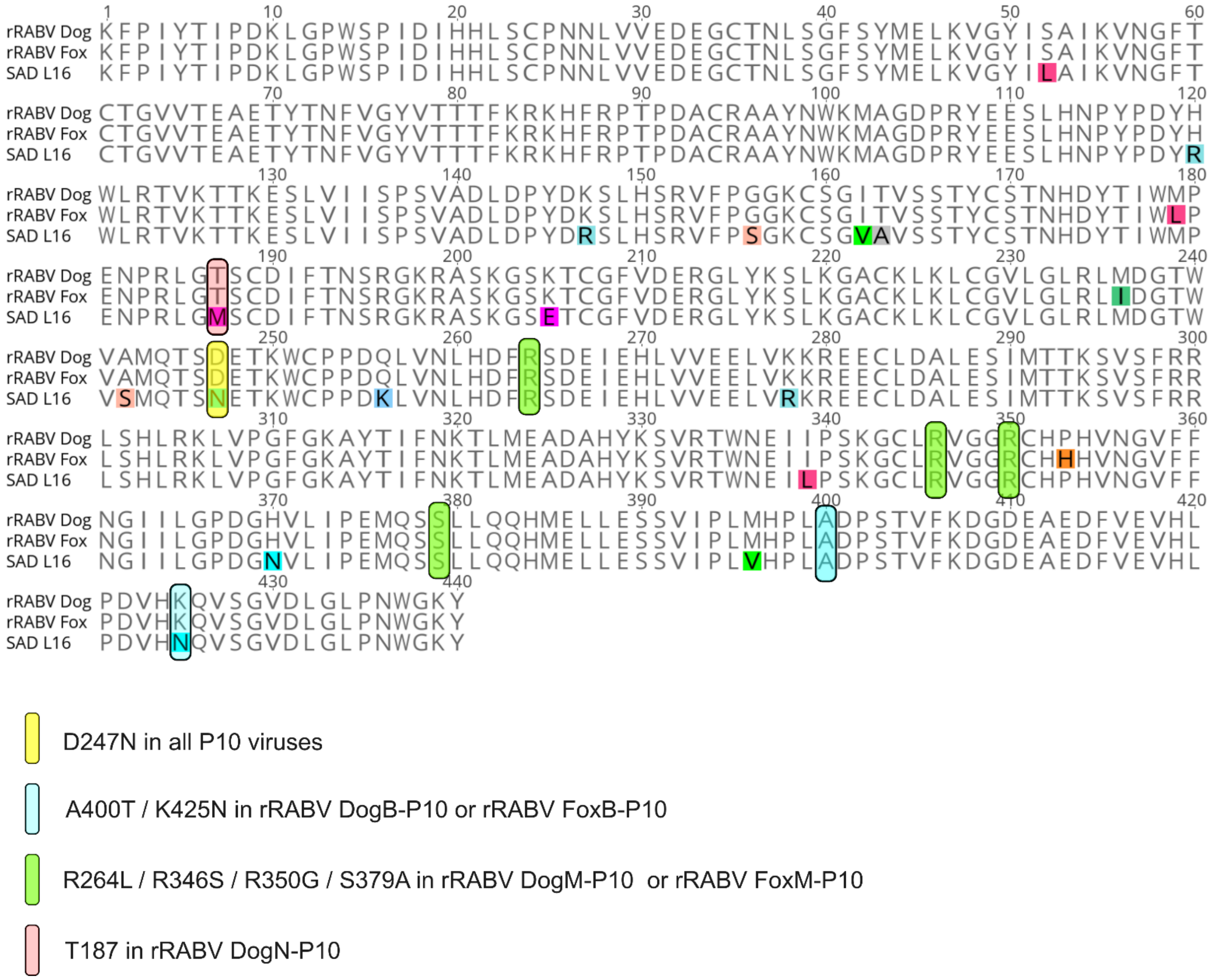

Figure S2: Comparison of the amino acid sequences of the rRABV Dog, rRABV Fox, and SAD L16 glycoprotein ectodomains. The attenuated SAD L16 sequence differed at 15 position from the rRABV Dog and rRABV Fox G sequences. rRABV Dog and rRABV Fox differed from each other at 3 positions. Amino acid positions associated with cell culture adaptation in the P10 viruses are indicated by coloured rectangles. Coloured squares indicate differences between authentic field virus clones rRABV Dog, rRABV Fox and SAD L16.
